# Subversion of the Immune Response by Rabies Virus

**DOI:** 10.3390/v8080231

**Published:** 2016-08-19

**Authors:** Terence P. Scott, Louis H. Nel

**Affiliations:** Department of Microbiology and Plant Pathology, University of Pretoria, Pretoria 0002, South Africa; terence.scott@up.ac.za

**Keywords:** rabies, immune evasion, influencing factors, therapeutics, apoptosis, rabies proteins

## Abstract

Rabies has affected mankind for several centuries and is one of the oldest known zoonoses. It is peculiar how little is known regarding the means by which rabies virus (RABV) evades the immune response and kills its host. This review investigates the complex interplay between RABV and the immune system, including the various means by which RABV evades, or advantageously utilizes, the host immune response in order to ensure successful replication and spread to another host. Different factors that influence immune responses—including age, sex, cerebral lateralization and temperature—are discussed, with specific reference to RABV and the effects on host morbidity and mortality. We also investigate the role of apoptosis and discuss whether it is a detrimental or beneficial mechanism of the host’s response to infection. The various RABV proteins and their roles in immune evasion are examined in depth with reference to important domains and the downstream effects of these interactions. Lastly, an overview of the means by which RABV evades important immune responses is provided. The research discussed in this review will be important in determining the roles of the immune response during RABV infections as well as to highlight important therapeutic target regions and potential strategies for rabies treatment.

## 1. Introduction

Rabies has afflicted mankind for centuries [1], and although there have been several significant advances towards the prevention and elimination of rabies—such as the development of the first rabies vaccine by Louis Pasteur [2,3]—relatively little is understood regarding the means by which rabies virus (RABV) infects, spreads, evades the immune system, and ultimately kills. We know that the majority of human cases result in death by circulatory insufficiency [4], but how does viral infection result in this outcome? In addition, how does RABV evade, or utilize, the immune system in order to successfully replicate and spread? In this review, we discuss the question of immune evasion and exploitation by addressing multiple aspects of the virus as well as the responses of the immune system to RABV infection. We provide a comprehensive summary and some of our own insights in order to highlight gaps or inconsistencies that may be addressed in future studies.

## 2. Experimental and Non-Experimental Factors Influencing the Immune Response

The innate and adaptive immune systems of mammals are closely linked, and response to an infection is dependent on a variety of factors.

### 2.1. Age

It is known that age influences the susceptibility of mammals to RABV infections, with young individuals typically more susceptible to infection than mature ones, irrespective of previous exposures [5,6,7]. This phenomenon has been demonstrated with both street and fixed strains of RABV, many decades ago. In these early experiments, seven-day-old mice were noted to be 10 to 1000-fold more susceptible to RABV infection when compared with 20- and 60-day-old mice, depending on the route of infection [8]. Similarly, dogs vaccinated at 11–16 weeks of age responded better than those vaccinated at 5–10 weeks [9]. In another study, both cats and dogs older than one year had better antibody responses than those younger than one year [10]. However, extended age results in a gradual decline in immune responses, as evidenced in dogs, cats and humans, resulting in differences in responses to vaccination [10,11,12]. Thus, depending on the age of the individual, differences in pathogenicity, immune responses, infection rates, survival times and mortality rates can be expected.

### 2.2. Timing

Rabies vaccines are designed to generate a protective neutralizing antibody response when administered a sufficient period prior to exposure. The same vaccine can also be administered post-exposure within a certain time frame in order to prevent the onset of symptoms. However, once symptoms have developed, the administration of vaccine to an individual is likely to be futile [13] or even detrimental [14]. If vaccine is administered close to the time of challenge, the host is more susceptible to infection due to a lower antibody response, compared with a longer interval between vaccination and challenge [7,10,12].

### 2.3. Sex

Sexual dimorphism can result in dissimilar immune responses to viral infection, most likely due to differences in hormones between the sexes. Sexual hormones, including estrogen, play an important role in immune-endocrine relationships that can affect the ability of the host to combat infection [15]. Typically, males are more susceptible to viral infections than females [15]. In rabies, a brief study demonstrated that female mice showed 6–12 times the efficiency in resisting challenge (Fixed virus, intra-cerebral (I.C.) challenge), as opposed to males, despite equal doses of vaccine and challenge virus among the genders [7]. Later studies confirmed these differences in mice challenged with varying doses of both fixed and street RABV strains [16]. Similarly in cats, antibody responses to vaccination varied significantly between sexes [10]. Furthermore, in humans, more females developed serum sickness compared with males when testing rabies immunoglobulin (RIG), emphasizing differences in immune responses among sexes [17,18].

### 2.4. Temperature

Temperature is an important factor in immunity, as non-optimal temperatures will result in the development of various stresses. Especially important is the consideration of temperature in animal models, where different animals require different ambient temperatures (particularly between periods of activity and inactivity), despite the fact that the animal may appear healthy at room temperature. In rabies research, the mouse model is frequently used, but mice are typically kept in room temperature (20–25 °C) housing. However, the optimal housing conditions for mice, especially during their inactive phase, are 30–32 °C [19]. Sub-optimal housing results in cold stress which subsequently affects the immune response [19]. Mice infected intraperitoneally with a street (V-16) or fixed (PV-1) RABV strain showed longer incubation periods, delayed onset of symptoms and lower mortality rates when the mice were housed optimally, as compared to being housed at room temperature [20].

### 2.5. Genetic Polymorphisms

Genetic differences among individuals, populations and strains of the host can result in different immune responses to different insults. This variation is due to the differential expression of innate immune genes among different strains exposed to the same insult, as only a small percentage of core genes are expressed among the various strains [21]. Genetic polymorphism among inbred strains of experimental mice after intraperitoneal challenge with a street RABV (5 × 10^7^ mouse intracerebral 50% lethal dose (MICLD_50_)) showed that some strains are highly resistant to infection, whereas others are highly susceptible, for example SJL compared to A/WySn, respectively [16]. Furthermore, the resistance of certain mouse strains was not attributable to the challenge virus used, as six different street RABVs were used as challenge viruses to the SJL (highly resistant; 100% survival) and the A.SW/Sn (highly susceptible; less than 20% survival) [16].

### 2.6. The Appropriate Animal Model and Other Influential Factors

There are a variety of other factors that also influence the immune system, including the diet of the host, circadian rhythms and the microbiota. Microbiota have been shown to play a role in infections outside of the digestive tract, for example, influencing the susceptibility to allergies in humans [22]. Despite the possible importance of these factors, there is to our knowledge no information related to RABV infections.

The route of inoculation in experimental models also has a significant impact on pathogenicity studies as well as the antibody responses associated with the challenges [23,24,25]. Typically, IC inoculation does not represent an accurate reflection of natural infections, but it does provide a good baseline from which the various other methods of inoculation can be compared and gauged [23,26]. Additionally, as IC inoculation bypasses the need for the virus to travel to the brain, incubation periods are typically shorter in animals inoculated in this manner than those inoculated peripherally [4,24]. The susceptibility of certain animal species to RABV infection is also influenced by the route of inoculation [27,28,29]. Therefore, the choice of an appropriate animal model can also be highly influential in studies of pathogenicity and immune response to infection. With regards to RABV, canines and chiroptera would be the most appropriate models for the viruses from these respective reservoirs. However, due to the ethical constraints as well as the specialized facilities required for housing these animals, murine models are often used in initial studies, despite the fact that mice are not natural reservoirs for RABV. Nevertheless, contrary to common perceptions, one study has shown that the innate immune responses between mice and raccoons (a natural reservoir) did not differ significantly [30]. In this study, innate responses did however occur at later time points when compared with a mouse model—but is likely due to the differences in the size of the animals used, as RABV travels at a relatively constant rate (12–100 mm/day) through the peripheral nervous system (PNS) and central nervous system (CNS) [31,32,33]. Yamaoka et al. (2013) [34] also observed that there were no innate responses at the site of infection, correlating with other observations that more pathogenic strains tend to suppress immune responses at the site of inoculation in order to replicate in the muscle and effectively enter the PNS. Significant upregulation of the innate genes under observation was observed in the brains of infected animals, but was only apparent when clinical symptoms presented. Contrarily, in the brains of animals that did not demonstrate clinical symptoms, immune gene transcripts were not significantly upregulated [30]. Despite all animals in that study being challenged under the same conditions (intramuscular (IM) inoculation, 10^6.7^ tissue culture infective dose (TCID_50_) raccoon rabies virus (RRV)), differences in morbidity and mortality were observed—leading the authors to consider genetic variation among the immune genes studied. Despite the variations in pathogenicity among animals, there were no significant genetic variations in the observed genes, suggesting that other factors play important roles in determining the success of the infection in the host [30].

### 2.7. Cerebral Lateralization

Cerebral lateralization is known to influence behavioral traits such as hand or paw preference, head turning and other behavioral biases [35,36,37]. Cerebral lateralization is prominent in a variety of animals, both domestic and wild [38,39], and it is thought to be a fundamental feature of all vertebrates [40]. In the majority of vertebrates, cerebral lateralization has a population bias due to social selection pressures [41]. The differences in cerebral lateralization result in different responses to various immunological stimulants, including responses to stresses and vaccination [42,43]. Sex has also been shown to be an important factor in cerebral lateralization and its effect on immune function, suggesting that sex hormones may be influential in the association between lateralization and immune function [44].

Cerebral lateralization not only affects the basal immune profile of the organism—such as total lymphocyte counts and total granulocyte counts [45]—but also affects responses to vaccinations and insults. In dogs vaccinated against RABV, a general basal increase in catecholamine levels was observed, but the increases showed a different kinetic profile depending on the lateralization characteristics of the animal [46,47]. Left-pawed dogs showed an increase in epinephrine early on after vaccination, but these levels returned to basal levels seven days after vaccination, whereas right- and ambi-pawed dogs showed a linear increase up until day 21 after vaccination [47]. Due to these differences in catecholamine levels, left-pawed dogs expressed lower levels of RABV neutralizing antibodies as well as lower levels of interferon-gamma (IFN-γ) when compared with right-pawed or ambidextrous dogs [47]. The influential or controlling effects of the sympathetic nervous system on the immune system can be explained by the fact that many immune cells express functional adrenoreceptors, enabling immune control via catecholamines such as norepinephrine and epinephrine [48]. These results show that cerebral lateralization can have a significant effect on immune responses, therefore, individual hand or paw preferences can result in a variety of inter-individual polymorphic results among a test group during a challenge or vaccination trial.

## 3. Rabies Entry into the Nervous System: The Use of Stealth

A key advantage of RABV is the ability to replicate in the CNS—enabling it to proliferate in an immunologically privileged area of the host [49,50]. However, typical entry of the virus into the host occurs via injection into muscle tissue or other peripheral tissues (usually via a bite), where the RABV glycoprotein (G) binds to the nicotinic acetylcholine receptor on the muscle [51,52]. This means that RABV is exposed to innate and adaptive immune responses. How then, is RABV able to gain entry into the PNS, and subsequently, the CNS?

**Figure 1:**
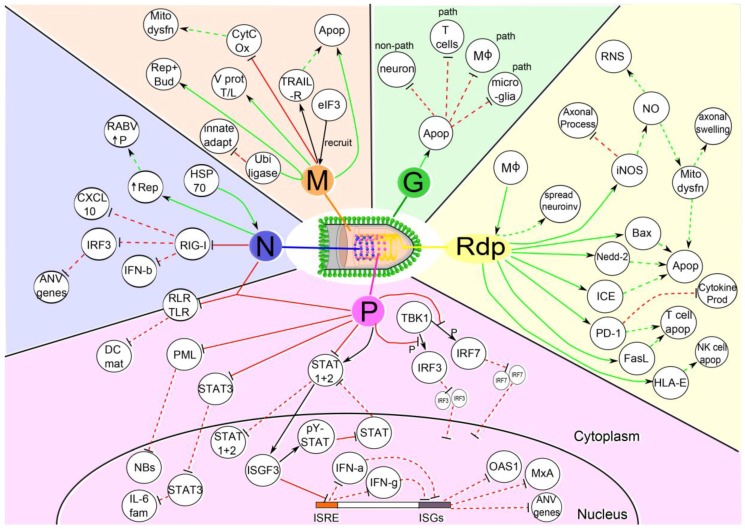
Overview of the various interactions between rabies virus proteins and immune factors. Interactions between rabies virus (RABV) and the immune system are depicted here. Each colored section refers specifically to the interactions between the main viral proteins and the host. RABV induces an upregulation of certain host processes in order to evade immune response and subsequently improve viral replication and spread (green arrows). Alternatively, RABV inhibits specific immune responses, resulting in increased pathogenicity and the evasion of downstream immune responses (red stop lines). Red lines indicate inhibition steps; Green lines indicate upregulation; Black arrows indicate interaction; Dashed lines indicate indirect influences/downstream interactions. Abbreviations: ANV genes, antiviral genes; Apop, apoptosis; Bax, Bcl-associated X protein; CXLC10, chemokine (C-X-C motif) ligand 10; CytC Ox, cytochrome C oxidase; Cytokine Prod, cytokine production; DC mat, dendritic cell maturation; eIF3, eukaryotic initiation factor 3; FasL, Fas Ligand; G, glycoprotein; HLA-E, human leukocyte antigen (HLA) class I histocompatibility antigen, alpha chain E; HSP70, heat shock protein 70; ICE, Interleukin-1b converting enzyme; IFN-a, interferon-alpha; IFN-b, interferon-beta; IFN-g, interferon-gamma; IL-6 fam, interleukin (IL)-6 family; innate adapt, innate and adaptive immune responses; iNOS, inducible nitric oxide; IRF3, -7, IFN regulatory factor 3, -7; ISGF-3, IFN-stimulated gene factor 3; M, matrix protein; Mito dysfn, mitochondrial dysfunction; MxA, myxovirus resistance-A; Mφ, macrophage; N, nucleoprotein; NBs, nuclear bodies; Nedd2, caspase-2; NK cell apop, natural killer cell apoptosis; NO, nitric oxide; non-path, non-pathogenic strains of RABV; OAS1, 2′-5′-oligoadenylate synthetase 1; P, phosphoprotein; path, pathogenic strains of RABV; PD-1, programmed death-1; PML, promyelocytic leukaemia; pY-STAT, tyrosine phosphorylated STAT; ↑ RABV P, increase in RABV P protein production; Rdp, replication-dependent processes; ↑ Rep, increased replication; Rep + Bud, replication and budding; RIG-I, retinoic acid-inducible gene I; RLR, RIG-I-like receptor; RNS, reactive nitrogen species; spread neuroinv, viral spread and neuroinvasion; STAT1–3, signal transducer and activator of transcription 1–3; TBK1, TANK-binding kinase 1; TLR, Toll-like receptor; TRAIL-R, TNF-related apoptosis-inducing ligand-receptor; Ubi ligase, Ubiquitin ligase; V Prot T/L, viral protein translation.

After the initial injection of the virus into the muscle tissues, RABV is able to undergo low levels of replication in the muscle tissue for a variable amount of time [34,53,54]. This period of low replication without subsequent spread possibly explains the variable incubation period of the virus during infection [25,52]. In cases of high titers of inocula, RABV is able to infect motor endplates without the need for the initial low-rate replication in the muscle [25,55]. Contrarily, low doses of inocula require a longer incubation period in the muscle before entering the PNS. How does RABV enter the PNS? The current dogma states that the virus in the muscle infects motor endplates and enters the PNS via the neuromuscular junction [25,51,52,56,57,58]. How then, does RABV evade the initial immune responses in the muscle and enter the PNS? Replication and entry into the PNS was shown to be dependent upon the specific virus strain, with more pathogenic strains exhibiting an increased efficiency for neuroinvasiveness [34,59]. The increased efficiency was not due to differences in replication efficiency or an increased ability to infect neurons, as these factors were comparable between the pathogenic and non-pathogenic strains. However, it was noted that RABV proteins played a role in sequestering the immune response, allowing for more efficient replication in muscle tissues. The sequestration was primarily dependent on the RABV phosphoprotein (P), but both the nucleoprotein (N) and the G protein also contributed [34]. The P protein was shown to specifically target the *interferon-beta* (*Ifn-β*), *myxovirus resistance protein 1* (*Mx1*) and *2′-5′-oligoadenylate synthetase 1* (*Oas1*) genes, resulting in the decreased expression of these genes that are important in innate responses against viral infections (Figure 1) [30,34]. Thus, the authors conclude that the efficient replication of RABV in muscle tissues is vital for its neuroinvasion and is dependent on the sequestration of specific immune responses in muscle tissues.

The immune response may also aid the dissemination of RABV throughout the PNS through the use of immune cells as vehicles. The Evelyn Rokitniki Abelseth (ERA) and Challenge virus standard (CVS) strains of RABV have both been shown to undergo active replication in activated lymphocytes (Jurkat T cells) in vitro, with a 10-fold greater replication rate in ERA-infected cells when compared with CVS [60]. Some RABV strains have been shown to be passively transported by dendritic cells (DCs) [61]. Despite being unable to replicate in DCs, both CVS and ERA strains of RABV were able to transfer infectious genetic material from the DCs to mouse neuroblastoma (MNA) cells in vitro, resulting in subsequent viral replication and proliferation in the MNA cells. The infectious genetic material from CVS stimulated neither type I IFN responses nor major histocompatibility complex (MHC) class I expression upregulation. In contrast, the ERA strain resulted in a more significant upregulation of both type I IFN production and MHC I expression. Similar results were obtained in vivo, where mice were injected with RABV-infected DCs, resulting in the development of symptoms and the subsequent death of the infected mice. The authors hypothesized that the migration of the mature DCs to the lymphoid tissues is possibly inhibited by the pathogenic RABVs, and that these mature DCs facilitate the spread of RABV into the neural cells of the PNS while evading a deleterious immune response [61].

CVS has been shown to be able to replicate in, and be released from, P388D1 macrophage-like cells [62]. More virus was released from the P388D1 cells in the presence of antiserum than in its absence, suggesting a further role of the immune system in the proliferation and spread of RABV. A second study demonstrated that ERA was unable to replicate in P388D1 macrophage-like cells, but infectious virus was present [63]. In the same study, RABV was isolated from macrophages 96 days post-infection, suggesting that RABVs may be able to persist in these cells [63]. Active RABV infection and replication within the macrophage cell lines only occurred in differentiated macrophage cell lines [63]. Furthermore, another report demonstrated that the CVS and high egg passage-Flury (HEP-Flury) strains of RABV were actively phagocytosed and unable to replicate in adult mouse macrophages, but macrophages from suckling mice were able to disseminate RABV in BHK-21 cells in vitro [5]. Thus, the age of the host and the maturity of the immune cells may play an important role in the dissemination and spread of RABV within the PNS. Despite the fact that the majority of these observations occurred in vitro, with limited in vivo evidence, it is unknown whether these occur in vivo in natural infections.

## 4. Apoptosis: Detrimental or Beneficial to the Host?

Apoptosis can be induced as a means to inhibit the replication and spread of viruses, or alternatively, it can be a mechanism used by viruses in order to avoid immune responses. Some viruses inhibit apoptosis [64], allowing uninterrupted replication within a cell, whilst other viruses replicate rapidly enough to spread before the apoptotic death of the cell [65]. Furthermore, some viruses trigger apoptosis in immune cells as an immune-evasive strategy, for example human immunodeficiency virus (HIV) [66]. Historically, apoptosis in RABV infections was thought to be solely induced by the expression of the RABV G protein—where the higher the levels of surface G protein, the higher the levels of apoptosis observed (see Figure 1) [60,67,68,69,70,71]. However, more recent work has shown that the G protein levels are not the only factor in influencing apoptotic events. The increase in G protein levels does not render a pathogenic virus apathogenic and similarly, the decrease in G protein levels does not render an apathogenic virus pathogenic; however, it does still have an influence on the pathogenicity of the virus [72]. Additionally, specific PDZ domain binding sites on the cytoplasmic domain of the RABV G protein interact with microtubule associated serine/threonine kinase 2 (MAST2) and protein tyrosine phosphatase, non-receptor type 4 (PTPN4) resulting in the control of neuronal apoptosis [73]. The RABV G protein from pathogenic and attenuated strains has different distribution patterns in neuronal cells, resulting in the anti- and pro-apoptotic abilities of the different strains, respectively [70]. The differences in G protein associations between the apoptotic and non-apoptotic strains may also be due to differences in the degradation rates of the G proteins—where G proteins of apoptotic strains degrade slower than those of the non-apoptotic strains [69,70,71].

Discrepancies between murine models and natural infections in dogs and humans have questioned the applicability of the studies in murine models [23], emphasizing the importance of the applicability of the correct animal model. However, it would appear that neuronal apoptosis occurs as a result of infection by less pathogenic strains of RABV, whereas apoptosis in immune molecules such as T cells, would be as a result of infection by a pathogenic strain of RABV, as evidenced below.

### 4.1. Neuronal Apoptosis as a Host Defence Mechanism

It has been hypothesized that programmed cell death is a host defense mechanism that occurs as a result of the immune response to RABV infection, acting rapidly in order to destroy infected neurons and limit the spread of the virus [68,69,74]. RABV-induced apoptosis in attenuated viral strains may also help in the presentation of antigen in cells infected with RABV [60].

However, in contrast to the hypothesis that apoptosis may limit viral spread, Purkinje cells of the cerebellum—a cell type typically targeted by RABV in order to replicate [75]—do not undergo apoptosis [76,77], or undergo limited apoptosis [68,78]. Further studies in humans have shown that neuronal apoptosis was not an important factor in RABV infection [23,79], with similar results in bats [80]. Despite the lack of apoptosis in neurons during bat experimental infections and natural RABV infections in dogs and humans, other studies have shown high levels of neuronal apoptosis in mice [6,69,76,77,78,81]. Many of the murine studies, however, were performed in suckling mice which, as discussed earlier, are more susceptible to infection due to the fact that their neurons have not fully matured [6]. The role of neuronal apoptosis is thus unlikely to be significant during natural RABV infections. However, cells infected with pathogenic strains do not undergo neuronal apoptosis but are rather affected by neuronal dysfunction, in contrast to the attenuated strains [56,58]. Pathogenic strains of RABV induce neuronal dysfunction, resulting in axonal and dendritic swelling and beading [58,82,83,84,85]. These morphological changes are thought to be sufficient to explain the onset of clinical signs and symptoms [83]. The neuronal dysfunction is caused by mitochondrial dysfunction that is affected by the RABV P protein as well as the matrix (M) protein in Mokola virus (MOKV) [58,85,86]. Therefore, differences between pathogenic and attenuated strains of RABV determine whether neurons undergo apoptosis as a host defense mechanism or whether neuronal dysfunction occurs, explaining the onset of clinical signs and symptoms.

### 4.2. Apoptosis as a Mechanism for Immune Subversion

Instead of being a host defense mechanism for the elimination of infected neurons, apoptosis could be specifically induced by RABV as an immune subversive strategy. Ultimately, RABV would aim to preserve neurons in order to facilitate rapid and undisturbed replication throughout the CNS, while concurrently hindering immune molecules—preventing viral clearance by the immune system. There have been observations of mild numbers of inflammatory cells, macrophages and oligodendrocytes, as well as a large number of infiltrating T cells, undergoing apoptosis in infected tissues during natural RABV infection [87,88,89], suggesting that these may be the targets of RABV-induced apoptosis. The inherent differences in pathogenicity between non-lethal and lethal strains of RABV may be due to the ability of non-lethal strains to induce apoptosis in neurons, as opposed to the lethal strains inducing programmed death in infiltrating T cells (Figure 1) [56,82,87,90,91], as fewer infiltrating lymphocytes have been detected in the brains of animals infected with pathogenic strains [92]. Similarly, HIV has been shown to sensitize T cells to tumor necrosis factor (TNF)-related apoptosis-inducing ligand (TRAIL)- and CD95(Fas/APO-1)-ligand (CD95L)-mediated apoptosis [93]. Interestingly, there is also a correlation between the pathogenicity of the RABV and the extent of neuronal apoptosis [82,84]. The correlation between pathogenesis and apoptosis is consistent with observations of apoptosis in inflammatory cells, microglia and macrophages in natural RABV infections in humans and dogs [23,79]. Therefore, non-lethal strains are cleared from the CNS by infiltrating T cells—despite evidence of RABV antigen in the brain—whereas lethal strains destroy these T cells and are thus free to replicate and spread throughout the CNS [56,87,90]. In support of this hypothesis, several apoptotic pathways are involved in RABV infections that have specific ligands and receptors directly involved with the destruction or inhibition of T cells.

### 4.3. Regulation of Immunosubversive Molecules Involved in Apoptosis

The Fas/Fas-ligand (FasL, CD95L) pathway is an important pathway in the maintenance of immune privilege in the host [94]. Immune privilege involves the destruction of inflammatory and infiltrating immune cells after injury or infection of immune privileged sites such as the brain and testes. This programmed death prevents immune responses from damaging the tissue due to excessive inflammation or cytotoxic responses after the insult has been resolved [95]. FasL is constitutively expressed by motor neurons in the CNS, and when Fas+ T cells bind to FasL, infiltrating T cells undergo apoptosis [94]. FasL is upregulated during lethal RABV infections and this stimulation correlates with viral progression through the CNS [49,91]. In contrast, in non-lethal RABV strains, FasL is only upregulated after viral clearance from the brain—likely as a result of normal immune privilege (Figure 1) [49]. Similarly, human leukocyte antigen (HLA)-G is also upregulated during RABV infection [95,96,97] and has also been suggested to play a role in the immunosubversive strategies employed by RABV in the CNS [95]. HLA class I histocompatibility antigen, alpha chain E (HLA-E) is also known to have a role in the destruction of natural killer (NK) cells, and all HLA molecules are up-regulated during RABV infection [96]. However, HLA-E is only expressed in the cytoplasm and is therefore thought to not play a significant, known role in RABV immune evasion [96], especially as NK cells are not known to be stimulated during RABV infection in humans [98]. Another pathway that is suspected to be involved in RABV immune-evasion is the programmed death-1 (PD-1) inhibitory molecule that is part of the B7-CD28 group. PD-1 has two ligands: B7 homolog 1 (B7-H1) and B7 dendritic cell molecule (B7-DC), and the binding of B7-H1 to PD-1 results in the inhibition of T cell proliferation and cytokine production [99]. RABV stimulates the expression of B7-H1 early in the infection of neurons, leading to the inhibition of cytokine production and T cell proliferation [91,100]. This inhibition was shown in B7-H1 deficient mice, where deficient mice survived longer than wild-type mice, and did not develop symptoms of encephalitis [100]. Although these pathways play important roles in apoptosis and in the lethality of these strains, they are not independently responsible pathways [95,96], suggesting that either these pathways play cooperative roles in infection and immune-evasion, or that there are still more molecules and pathways that interplay for a successful RABV infection and subsequent immune-evasion.

### 4.4. Caspase-Dependent and -Independent Apoptotic Pathways

Through the upregulation of various apoptotic cascades and factors, apoptosis can be induced via multiple different pathways, complicating the potential for treatment or intervention. Several caspase-dependent apoptotic factors are involved in RABV-induced apoptosis. The Bcl-associated X protein (Bax) is a member of the Bcl-2 family of proteins that are involved in the positive and negative regulation of programmed death. Bax is a positive regulator of apoptosis, whereas Bcl-2 is a negative regulator, thus making the ratio between Bax and Bcl-2 an important determinant for caspase-dependent apoptotic induction. RABV induces the upregulation of Bax protein, while the levels of Bcl-2 remain unchanged [101]—leading to the induction of apoptosis (Figure 1) [77,102]. The Bax protein was shown to be important in the induction of apoptosis during RABV infection, but not essential [76], suggesting that yet other apoptotic regulators are involved in RABV infection. Other caspases such as caspase-1/IL-1b converting enzyme (ICE), caspase 2/Nedd-2, caspase 3 and caspase 8 are also upregulated during RABV infection [103]. ICE has been linked to nitric oxide (NO) production via inducible nitric oxide synthase (iNOS) [101], while Nedd-2 is a developmentally downregulated neuronal apoptotic gene that is upregulated by RABV in adult mice [104]. There is also evidence for the presence of caspase-independent pathways of apoptosis, as one study showed that there were fewer neurons positive for caspase-3 than there were neurons undergoing apoptosis [78]. Additionally, more caspase-3 activity was noted in suckling mice when compared with adult mice, showing another difference in the susceptibility and the pathways utilized in the different age groups considered [6,78]. Evidence also suggests that the M protein is involved in TRAIL-mediated apoptosis via the extrinsic pathway, with caspase-8 being principally involved and leading to the activation of caspases-3 and -6 (Figure 1) [105,106].

## 5. Contributing Factors and Mechanisms to RABV Pathogenesis

During RABV infection of the CNS in a murine model, more than 390 genes are differentially expressed in comparison to mock-infected control mice [107]. The majority of these genes are involved in innate immune responses and host defense mechanisms including the Janus kinase/signal transducers and activators of transcription (JAK/STAT) pathway and antigen presentation [107]. Several of these factors play a variety of roles in normal cellular function as well as in antiviral responses. RABV uses some of these factors in its own immune-evasive strategies.

### 5.1. Nitric Oxide

In normal, physiological amounts, NO is an important gaseous molecule involved in the homeostatic functions of mammalian hosts [108]. It has an especially important role in the CNS where it is involved in hormone secretion, the sleep-wake cycle, regulation of body temperature and synaptic plasticity [108]. Without these low levels of NO in the CNS, the host would not be able to function. NO is also involved in immune responses and other essential functions throughout the body, including peristalsis in the gastro-intestinal tract and smooth muscle function [108].

Nitric oxide is produced from l-arginine by NOS and constitutive NOS (cNOS) is responsible for the physiological levels of NO production in neurons and endothelial cells [101]. Higher levels of NO, however, can be produced through the induction of iNOS. The expression of iNOS is typically induced by inflammatory cytokines or endotoxins [101] and leads to higher levels of NO, resulting in the formation of reactive nitrogen species (RNS) which are harmful to the cell [108]. RNS have been implicated in the progression and pathogenesis of neurodegenerative disorders such as amyotrophic lateral sclerosis (ALS) [109] and more recently in viral infections.

Nitric oxide has been implicated in the immune response against RABV as well as in its pathogenesis. The artificial generation of low levels of NO showed that NO was able to inhibit RABV replication and viral protein synthesis in vitro [110]. Contrarily, several other studies have shown that RABV induces the production of iNOS in neurons and macrophages, which leads to increased pathogenesis and the hypothesized explanation of symptoms such as encephalitis as well as symptoms in other non-neuronal organs [101,111,112,113,114,115,116,117]. The production of NO by iNOS has been implicated in neuronal apoptosis in some studies—suggesting that neuronal apoptosis was either the cause of the pathology observed in RABV infections or as a result of unadulterated RABV replication [101]. However, later studies have shown that rabies symptoms are as a result of neuronal dysfunction rather than neuronal death [74,82,83,84,118,119]. High levels of iNOS production by neuronal cells and microglia leads to axonal swelling—a pathology that has been closely correlated with the onset of symptoms, including impaired axonal transport of proteins and subsequent axonal degeneration (Figure 1) [114]. Furthermore, in vitro studies using dorsal root ganglion (DRG) cells have shown that CVS was unable to cause a loss of viability of DRG neurons, but was able to inhibit the growth of axonal processes [114]. Axonal swelling and apoptotic cell death in neurons are likely a result of mitochondrial dysfunction [86,114].

Nitric oxide is an important molecule in innate immunity, however, RABV has adapted to stimulate up to 10 times the homeostatic levels of NO in infected neurons and macrophages [111]. This dramatic increase in NO is toxic to the cell and can result in apoptosis and mitochondrial dysfunction with detrimental resultant effects, such as axonal swelling. The resultant effects of high NO levels has been implicated in the development of the encephalitis [111,120] as well as possible non-neuronal organ pathologies [113], likely revealing how RABV infections result in the death of the host.

### 5.2. Mitochondrial Dysfunction

Pathogenic strains of RABV have been shown to induce mitochondrial dysfunction in neuronal cells during infection, resulting in axonal and dendritic swelling and beading [83,84,85,121]. Mitochondrial respiratory chain complex I activity is significantly upregulated in CVS infected neuronal cells, as is complex IV activity. However, the upregulation of complex I activity is closely correlated with the susceptibility of different neuronal cell types to RABV infection, whereas complex IV activity correlates less closely [121]. Alandijany and colleagues showed that CVS infection resulted in an enhanced mitochondrial membrane potential which favors ROS production [121]. Additionally, ATP levels in the CVS infected cells were diminished—likely as a result of the presence of ROS, as hydrogen peroxide is known to reduce cellular ATP levels in a time- and dose-dependent manner [121,122]. Mitochondrial dysfunction induced by CVS resulted in an increased production of ROS as complex I is considered one of the main ROS generators [85,121,123]. The P protein of RABV was determined to be the protein involved in direct interactions with the mitochondria and is therefore the protein responsible for mitochondrial dysfunction in RABV infection. More specifically, amino acids 139–172 of the RABV P protein were shown to be important in its direct interaction with the mitochondrial complex I [85]. We speculate that the interactions of the P protein with STAT3 are essential for the interactions of P with the mitochondrial complex 1, as STAT3 can regulate mitochondrial function, depending on its phosphorylation status [85,124]. Furthermore, inhibition of the STAT3 results in the decrease in complex I activity and it has been shown that STAT3 signaling is inhibited by RABV P protein [125].

Interestingly, during RABV infection, some ROS generation continued in the presence of a complex I inhibitor, suggesting that ROS generation occurs at sites other than complex I. This supports other studies that indicated that during CVS infection, the P protein of RABV played a pivotal role in mitochondrial dysfunction, whereas the M protein of MOKV was responsible for these effects, via a different mechanism [85,86]. In RABV infection, M protein significantly reduced the activity of complex I and was not present in any mitochondrial extracts whilst during MOKV infection, the M protein inhibited complex IV activity [85,86]. Thus, different lyssaviruses induce mitochondrial dysfunction in different manners, with the aid of different viral proteins, suggesting that these redundancies are essential in lyssavirus infections. Mitochondrial dysfunction results in neuronal dysfunction and the impairment of neuronal processes. This impairment has been implicated in the development of clinical signs during RABV infection which are essential for the spread of the virus to another host.

### 5.3. Heat Shock Proteins

Heat shock proteins (HSPs) are involved in a variety of normal cellular functions including signal transduction and cell cycle regulation, as well as apoptosis and innate immune responses [126,127]. HSPs have also been shown to be involved in the replication process of several viruses, as protein folding can become a limiting step. Thus, viruses recruit HSPs as chaperones for their own protein folding, enabling efficient viral replication. Heat shock protein 70 (HSP70) has been shown to be involved in RABV replication and has been identified within RABV virions (Figure 1) [128]. HSP70 is upregulated—as early as 4 h post-infection—during RABV viral replication and has been shown to bind specifically with the RABV N protein in the nucleocapsid (Figure 1) [128]. When HSP70 was overexpressed, a notable increase in P protein synthesis occurred, and overall viral production was also increased. However, a reduction in HSP70 led to the dose-dependent decrease in viral production—including a three to eight-fold reduction in N and P proteins—without any inhibition of the production of viral mRNA, thus implying that HSP70 has a proviral effect during RABV replication [128].

### 5.4. Glucocorticoids

Glucocorticoids are steroids that are important in homeostasis as well as in immune regulation. They have specific roles in attenuating inflammatory responses in order to prevent damage caused by overt inflammatory reactions due to an insult, and are regulated primarily by the hypothalamic-pituitary axis (HPA) [129]. In a RABV challenge trial with a pathogenic street strain, corticosterone levels were shown to be 12-fold higher than those of mock-infected animals [130]. This result was shown to be independent of the stresses induced by the injection and handling of the experimental animals. The greater levels of corticosterone correlated with an increase in viral titers that can be explained by the fact that corticosterone inhibits inflammatory responses that are essential for viral clearance and antigen presentation. Supporting this, the use of a glucocorticoid inhibitor resulted in reduced mortalities and clinical signs [130].

Several other studies have shown the negative effects of glucocorticoid treatment during RABV infection, including the complete inhibition of virus-neutralizing antibody (VNA) production after post-exposure prophylaxis (PEP) supplemented with cortisone in the case of a human rabies exposure [131]. The use of corticosteroids in a PEP setting has been shown to decrease the incubation period and increase the mortality rate in a variety of animal models [13,132,133], and corticosteroids have been used as intentional immune-suppressants for oral vaccine trials in foxes [134]. Additionally, late treatment with cortisone has been shown to diminish T cell responses in mice [135]. Thus, the stimulation of the HPA that results in the induction of glucocorticoids during RABV infection is an immunosuppressive mechanism used by RABV that results in the inhibition of an inflammatory response and the efficient presentation of antigen. The suppression of the inflammatory immune responses results in a greater viral load and a reduction in the production of VNAs, enabling rapid progression of the disease within the host.

## 6. The “Early Death” Phenomenon

The immune system is typically deemed as a beneficial defense mechanism against invading pathogens or insults. However, the immune system can also be inadvertently detrimental to the host and oft-times can be more detrimental to the host than the initial insult.

It is understood that VNAs typically confer protection against RABV challenge, and therefore, the presence of sufficient numbers of VNAs has been a reliable measure of protection after vaccination. Contrarily, B cells have been shown to play a role in the ‘early death’ phenomenon in RABV infection [62,136], while T cells may possibly prevent such a result [137]. Early death is a phenomenon where insufficient protection by antibodies—typically after vaccination—can lead to the earlier death of a host after challenge as compared to no vaccine controls. This phenomenon was first observed with regards to rabies in vaccine trials on monkeys in 1971 [138]. Later studies investigated early death through experimental trials in mice and cell culture models [62,136,139]. In one such example, 50 mice were challenged with CVS three days after their inoculation with either a French national reference preparation (FNRP) of rabies vaccine, or alternatively, a placebo. Six days after challenge, 42% of mice in the FNRP group had died, whereas none of the mice in the placebo group died. On day 7, 98% of the placebo mice survived whereas only 20% of the FNRP mice had survived [140]. A more recent survey of case studies has shown possible instances of early death in humans which has implications regarding the use of vaccine and RIG after the development of symptoms in human cases [14]. More specifically, early death is suggested to occur more frequently in bat rabies cases, in contrast to those of dog rabies cases in humans. However, limited human data precludes any definitive conclusions as to the effects of early death in humans [14]. The early death phenomenon has also been observed in other viral infections such as feline infectious peritonitis virus (FIPV) and yellow fever virus (YFV) [141,142]. The early death phenomenon once again emphasizes the importance of influential factors on immune responses and their effects during infection.

## 7. How Does RABV Do It?—the Mechanisms by Which RABV Sequesters the Immune System

### 7.1. Phosphoprotein

The RABV P protein is able to interfere with the IFN signal transduction pathway in three separate ways, leading to a weakened innate and adaptive immune response to infection (Figure 1). Interestingly, the inhibition of the IFN signal transduction pathways is dependent on the specific RABV P protein involved, as attenuated strains of RABV tend to have a weaker inhibition effect when compared with the virulent street strains, resulting in a direct correlation between the pathogenicity of the RABV strain and its ability to sequester the immune response [34,143,144].

#### 7.1.1. Interaction of Phosphoprotein and Nuclear Bodies—Effects on Antiviral Responses

Promyelocytic leukaemia (PML) protein is an IFN (type I and II) induced protein that typically localizes into structures called nuclear bodies (NBs). These NBs have been shown to play important roles in normal host cell function—such as p53-dependent apoptosis and transcription regulation [145]—as well as in antiviral defense against DNA viruses and cytoplasmic-replicating RNA viruses [146,147]. The RABV P protein C-terminal domain directly interacts with the really interesting new gene (RING) finger motif of PML. Interestingly, the truncated P, P3—derived from a ribosomal leaky scanning mechanism [148]—also interacts directly with PML leading to its co-immunoprecipitation in the cytoplasm [145,149,150]. The interaction between P and PML leads to a striking difference in NB size and structure when compared with normal NBs, with no detectable effects on NB numbers and distribution [149]. The sumoylated (small ubiquitin-like modifier) PML IV isoform has specific antiviral effects against RABV as it influences viral protein synthesis [150]. In PML^−/−^ cells, viral expression was 200-fold higher than that in wild-type mouse embryo fibroblasts [150], and this effect was shown to be significant in an IFN-independent manner. IFN, however, was still active in PML^−/−^ cells, albeit with a lower efficiency, suggesting that PML also plays an important role in IFN-induced immunity to RABV infection [150]. Thus, by influencing the localization and structure of the NBs, RABV P protein is able to sequester the antiviral effects of PML, as well as reduce the efficacy of type I and II IFN responses to infection.

#### 7.1.2. The Importance of the Nucleocytoplasmic Shuttling Abilities of RABV P Protein

The STAT proteins are important in IFN signal transduction pathways, in both type I and type II IFN responses. Briefly, after interferon-alpha (IFN-α) activation, IFNs bind to their receptors on cell surfaces and subsequently activate the JAK/STAT signaling pathway. The STAT proteins are phosphorylated by JAK, followed by the formation of homo- and hetero-dimers in IFN-γ and IFN-α responses, respectively [151]. The dimers subsequently bind to IFN regulatory factor 9 (IRF-9), resulting in the formation of the IFN-stimulated gene factor 3 (ISGF3) complex. ISGF3 translocates from the cytoplasm to the nucleus and binds to DNA containing the IFN-stimulated response element (ISRE) sequences, leading to the expression of a variety of antiviral gene products such as myxovirus resistance-1 (MxA), OAS1 and other IFN-stimulated gene (ISG) products [151].

Despite the fact that all lyssaviruses, and many other RNA viruses, replicate solely in the cytoplasm, many of these viruses encode proteins that have nucleocytoplasmic shuttling capabilities. In the case of RABV (and all of the other known lyssaviruses to date), the P protein has been shown to have nucleocytoplasmic shuttling capabilities due to the presence of an N-terminal nuclear export signal (NES) and a C-terminal nuclear localisation signal (NLS) [152,153]. Thus, the full-length P and P2 have a cytoplasmic distribution as it appears that the NES is able to override the effects of the NLS [152], whereas the N-terminally truncated P3, P4, and P5—derived from ribosomal leaky scanning [148]—have a nuclear localization. The NES contains a leucine-rich motif vital for its effective function, and this motif seems to be conserved among the RABV street strains as well as other lyssaviruses including MOKV and Lagos bat virus (LBV) [152,153].

What then, is the function of the nucleocytoplasmic shuttling capabilities of RABV P? The shuttling ability of RABV P has two important functions with regards to the inhibition of IFN signaling and responses. Firstly, it has been shown that the C-terminal domain (amino acids 268–297) of RABV P interacts directly with tyrosine phosphorylated STAT (pY-STAT) [154], leading to co-immunoprecipitation of the two proteins after IFN activation of the cell [153,154,155]. The P protein likely only interacts with pY-STAT proteins as this would ensure that cells that are not IFN-stimulated would function normally, as unphosphorylated STAT plays important roles in normal cellular functions [125]. Specifically, amino acids W265 and M287 of P are important in strong STAT binding and antagonism, as demonstrated by the fact that amino acid substitutions at these positions greatly inhibited the P-dependent antagonism on the IFN-dependent signaling and activation ofIFN-induced antiviral responses [156]. In light of this interaction between P and STAT, RABV is able to inhibit the IFN signaling cascade by preventing the nuclear localization of STAT. The ability of RABV to maintain STAT in the cytoplasm is dependent on the functioning of the NES signal as described above, where RABV strains with a fully functional NES are able to prevent IFN signaling [157]. Thus, it has been further demonstrated that the ability of RABV P to prevent the nuclear localization of STAT can be directly correlated with the pathogenicity of the RABV. RABVs that are able to maintain STAT in the cytoplasm have been shown to be more pathogenic than those that are unable to; for instance, the Nishigahara strain has a fully functional NES domain and its P is able to efficiently prevent the nuclear localization of STAT, thus inhibiting the subsequent IFN-dependent antiviral response [157]. However, the attenuated derivative strain Ni-CE has a two-amino acid substitution within the leucine-rich NES, resulting in a 500-fold greater sensitivity to IFN [157] and a marked decrease in pathogenicity [34,143,144].

STAT3 signaling has also been shown to be inhibited by RABV P in a similar manner to that of STAT1 and -2. The last 30 C-terminal amino acids of P are responsible for the binding of STAT3 and the prevention of its nuclear accumulation [125]. In contrast to STAT1 and -2 signaling, STAT3 is involved in the Gp130-receptor-dependent signaling leading to the activation of other immune molecules such as the interleukin (IL)-6 cytokine family [158]. Thus, the inhibition of the nuclear localization of pY-STAT3 results in a diminished IFN-stimulated cytokine response [125].

In addition to the full-length P protein, P2 has also been shown to be able to inhibit STAT signaling via the JAK/STAT signal transduction pathway, as it is also cytoplasmically located [148]. Therefore, it has been hypothesized that the function of the truncated P2 is predominantly focused towards IFN signaling inhibition, thus enabling the full-length P to be involved in the viral replication process [159].

Secondly, the full-length P and the truncated P3 are able to prevent the binding of ISGF3 and pY-STAT1 to the ISRE and gamma-activated sequence (GAS) promoters for IFN-α and -γ signaling, respectively, resulting in the inhibition of *ISG* expression in a dose dependent manner [160]. This inhibition occurs entirely in the nucleus, and is a result of the inhibition of the binding of STAT1 to the DNA promoters [160], through the direct interaction of the last 10 amino acids of P and P3 with STAT in the nucleus [154]. This specific mechanism of the inhibition of an effective antiviral response is thought to be independent of the ability of RABV P to inhibit the nuclear localization of STAT [160]. RABV P is also able to inhibit the dephosphorylation of pY-STAT in the nucleus, thus preventing the interaction between STAT and nuclear phosphatases resulting in the inhibition of the recycling of STAT for further signaling [153].

#### 7.1.3. RABV P Protein Impairs Phosphorylation of Interferon Regulatory Factor 3 and 7 

Interferon regulatory factor 3 (IRF3) is an important factor in initiating a type 1 IFN response, and is constitutively expressed in the cytoplasm of most cell types. IRF3 is phosphorylated by TRAF family member-associated NF-kappa-B activator (TANK)-binding kinase 1 (TBK-1) at the serine residue at position 386 of IRF3 as well as by I-kappa-B kinase epsilon (IKKε), resulting in the dimerization of IRF3 and its subsequent recruitment to the IFN-β enhancer as part of a protein complex [161]. IRF3 is able to function in an autocrine or paracrine manner and the resultant effect is the activation of IFN-α and/or -β.

RABV is able to specifically inhibit the activation of IRF3, IRF7 and IFN-β in addition to interfering with other IFN-related signaling pathways and PML nuclear body structures. The RABV P protein as well as the truncated P1 are able to directly inhibit the phosphorylation of IRF3 by TBK-1 at S386 [162] as well as IRF7 through interaction with amino acids 176–186 of the RABV P protein [52,163]. In vivo studies showed that a Street Alabama Dufferin (SAD) mutant with a deletionin P at amino acids176–181 was completely attenuated in mice, despite the fact that the mutant RABV was still able to efficiently interfere with STAT signaling and localization [163]. The lack of phosphorylation of IRF3 and 7 prevents their dimerization and therefore inhibits their nuclear localization, thereby preventing IFN-β transcription. Thus, the RABV P protein is able to inhibit IRF3 dimerization and subsequent innate and adaptive immune responses—most likely via a direct interaction with IRF3—resulting in a dampened host response to infection. The inhibition of IRF3 phosphorylation was shown to be effective in a dose-dependent manner, where a modified SAD strain expressing low amounts of P was unable to prevent IFN-β production, in contrast to the wild-type strain that abolished IFN-β expression [162].

#### 7.1.4. Indirect Consequences of P Protein on the Innate and Adaptive Immune Response

Dendritic cell maturation has been shown to be dependent on the RIG-1-like receptor (RLR) signaling pathway during RABV infection. Despite the fact that type I IFN is initially produced, autocrine signaling of IFN via IFN- α/β receptor (IFNAR) is essential for immature DCs to undergo maturation during RABV infection [164]. The autocrine feedback loop is dependent on the JAK/STAT signaling that upregulates the production of proteins required for type I IFN induction. However, the RABV P protein has been shown to specifically inhibit the JAK/STAT signaling cascade. This means that the more pathogenic strains of RABV will inhibit the maturation of DCs, as well as the production of antiviral genes. Furthermore, in the absence of IFN-α and -β induction, viral replication in DCs occurs at a more rapid rate—increasing the pathogenicity of the virus [164]. DCs play a pivotal role in both the innate and adaptive immune responses, and have been suggested to be an important part of the generation of a protective immune response following oral immunization [165]. Thus, the maturation of immature DCs is important in both innate and adaptive responses and is inhibited by the RABV P protein. This is supported by the fact that attenuated strains of RABV induce much higher levels of IFN-α/β levels than pathogenic strains [165].

### 7.2. Nucleoprotein

The nucleoprotein forms part of the ribonucleoprotein (RNP) complex and is involved in the encapsidation of the genomic RNA. It has been shown that the N-terminal domain (NTD) and the C-terminal domain (CTD) of N clamp down on the genomic RNA strand and enclose it completely [166]. The complete encapsidation is hypothesized to protect the RNA from recognition by the innate immune system of the host—more specifically recognition by Toll-like receptors (TLRs) and RLRs [166]. Furthermore, the N protein has a vital role in the suppression of the innate immune response during infection, allowing for increased viral replication and spread in the brain and CNS of the infected individual [167,168,169].

The N protein has been shown to be an efficient inhibitor of RIG-I activation (Figure 1). This was shown both in vivo and in vitro and was not related to the specific expression of viral genomic RNA. In a series of studies performed by Masatani and colleagues [167,168,169], the N gene from the pathogenic Nishigahara strain was inserted in place of the N gene in the attenuated Ni-CE strain, resulting in the formation of CE(NiN). The CE(NiN) and Ni strains showed greater inhibitions of the innate immune response—specifically IFN-β and chemokine (C-X-C motif) ligand 10 (CXCL10)—when compared to the attenuated wild-type Ni-CE strain. The inhibitory effect of the Ni N protein was attributed to the inhibition of the activation of RIG-I, resulting in the suppression of IRF3 as well as downstream antiviral genes. The effects of the N protein inhibition of RIG-I activation were shown to be independent of the effects of the P protein in the inhibition of STAT1 and 2 and the nuclear translocation and dimerization of IRF3. Thus, the N protein is an important contributing factor to innate immune evasion and suppression by RABV, resulting in increased viral replication in the brain and CNS and increased morbidity and mortality rates in vivo.

### 7.3. Matrix Protein

The primary function of the M protein is the recruitment and condensation of the ribonucleoprotein complex to the cellular membrane during replication, and the subsequent budding of the enveloped virus from the cell [170]. However, as with the majority of viral proteins, the M protein has several other functions that enable the efficient and successful replication and spread of RABV (Figure 1). One such example is the ability of the M protein to interact with eukaryotic initiation factor 3 (eIF3) in order to aid in the “hijacking” of host translational machinery [171]. The M protein has also been implicated in apoptotic events. It has been hypothesized that the late budding domain of the M protein works in conjunction with ubiquitin ligases in order to facilitate viral replication and efficient budding [172], with ubiquitin ligases being important factors in innate and adaptive immune response [173]. This suggests that RABV redirects resources from the immune response in order to facilitate its own replication and spread in the host.

### 7.4. Inhibition of the Host’s Immune Response by RABV—The Bigger Picture

In the early stages of infection, the RIG-I pattern recognition receptor (PRR) is able to recognize the 5′-triphosphate single-stranded RNA (ssRNA) of RABV, resulting in the initiation of a signaling cascade and an innate immune response. However, RABV has developed the means to inhibit this signaling cascade at several different steps, resulting in a host that is more susceptible to infection due to an inhibition of the innate and subsequent adaptive immune responses. The multifunctional abilities of each of the RABV proteins enable the inhibition of innate and adaptive immune responses at a variety of steps during immune detection and signaling. The seemingly redundant sequestration of various immune signaling and activation steps function independently from one another, and are especially important in the inhibition of IFN-related responses from a variety of signaling cascades. The supposed redundancy is critical in the inhibition of the initial activation, as well as the feedback loop regulation, of the IFN response through autocrine and paracrine signaling.

After the infection of a cell, RIG-I typically recognizes the RNA of the viral intruder. However, in the case of RABV, the viral RNA is protected from RIG-I detection, supposedly due to the encapsidation of the genomic RNA by the RABV N protein. Thus, RABV is able to inhibit detection by PRRs and thus prevents all further downstream responses to infection. If the 5′-triphosphate ssRNA binds to RIG-I, a signaling cascade involving the activation of IKKe and TBK-1 commences, resulting in the phosphorylation of IRF3 and -7, however, RABV P protein is able to inhibit the phosphorylation of IRF3 by TBK-1 as well as the phosphorylation of IRF7. Next, phosphorylated IRF3 and -7 translocate to the nucleus, and in a complex with other transcriptional co-activators, results in the activation of IFN-β. IFN-β then activates IFNAR in an auto- and paracrine manner. The binding of IFN-β to IFNAR results in the activation of JAK, consequently resulting in the phosphorylation of STAT-1, -2 and -3 and their dimerization. The dimerized STATs are then translocated to the nucleus where they bind to ISGF3 (STAT-1 and -2) or Gp130 (STAT3) and initiate antiviral gene expression or cytokine production, respectively. RABV P, as well as the truncated P1, is able to prevent the nuclear localization of the pY-STAT proteins, resulting in their cytoplasmic accumulation and the inhibition of antiviral and cytokine gene activation. Additionally, RABV P protein is able to inhibit the binding of ISGF3 to ISREs on the promoters of antiviral genes in an independent manner to the inhibition of the nuclear translocation of STAT.

Once an infection has been established and the virus has travelled via anti-retrograde transport to the CNS, the adaptive immune response becomes the primary means for viral clearance. RABV is able to inhibit the infiltration of T cells and other immune cells into the brain by inducing apoptosis in these cells. This then enables the pathogenic strains of RABV to establish an irreversible infection leading to the classically observed symptoms—through mitochondrial and subsequent neuronal dysfunction—and the inevitable fatal outcome of the disease.

## 8. Conclusions

In summary, RABV is able to sequester a variety of initial innate immune responses in the early stages of infection, which ultimately leads to the sequestration or hindering of any subsequent adaptive immune responses. Additionally, RABV is able to directly interfere with, and beneficially alter, adaptive immune responses later in infections in immune privileged areas such as the brain. RABV therefore targets a wide range of immune responses in order to ensure its successful and efficient replication in the infected host.

Many aspects with regards to how RABV replicates and causes the characteristic symptoms observed in typical infections and the specific mechanisms for many of the immune-evasive strategies that RABV uses, are still not understood. A better understanding of these strategies and the process by which RABV causes symptoms and death will lead to improved therapeutics and new potential therapeutic targets. There has been a dramatic increase in the number of new and developing viral therapeutic options including RNA-based therapies such as RNA interference (RNAi), aptamers and microRNA (miRNA)-based therapies. RNAi has thus far only been applied in a limited number of studies with regards to rabies [174,175,176,177,178,179,180], but has shown great potential, both within rabies, as well as in other viral diseases where research is at a more advanced stage [181]. However, several challenges still remain, including that of identifying the ideal targets for knockdown and the means of targeted delivery. Aptamers have been shown in other viral diseases to be exemplary delivery vehicles for targeted therapeutic delivery, yet have only thus far been used in rabies for direct inhibition of infected cells [182]. Additionally, in recent years, an improved understanding of miRNAs and their vital function in normal cellular function has become evident, with their influence upon RABV infection only beginning to be uncovered [177,183,184]. With an improved understanding of the immune system, as well as the means by which it is manipulated by RABV infection, these novel, developing therapeutic options should focus on targeting the critical genes involved in immune evasion, such as the P protein, as well as looking to control the immune responses that stimulate viral propagation and infection. By incorporating effective RNAi with specific, targeted viral gene knockdown and improved delivery mechanisms, these novel therapeutic options may be able to provide a cheaper, more easily produced and more available therapeutic option to those that currently exist for rabies. Following the success and progress made with other viral diseases, new rabies viral therapeutic options can rapidly progress and advance to the stage where they can be implemented in endemic countries to facilitate the progress towards canine-mediated human rabies elimination before the target date of 2030.

However, with the global target for canine-mediated human rabies elimination set for the year 2030 [185], the need for improved therapeutics and an increased understanding of rabies has been questioned. Exemplifying the doubt for further in-depth research into alternative therapeutic options, smallpox has been eradicated globally, yet the pathogenesis of the disease is still not completely understood [186]. This lack of understanding had no influence upon the eradication of the disease as an effective vaccine and intensive eradication plan were available, as is the case with rabies. However, we propose that although the global target of canine-mediated human rabies elimination by 2030 is feasible and on course to achievement, the spread of RABV and the other rabies-related lyssaviruses will continue in wildlife reservoir species. In recent years, it has been noted that new and emerging reservoir species for RABV have become evident and more problematic, thus posing a further risk to the human population. This has been most notable in raccoon and skunk rabies in North America [187,188], but is supported by the emergence of variants in other host species such as kudu in Namibia [189], among others, and the latest evidence of a natural infection of rabies in an avian species [190]. Furthermore, the increasing trend of the discovery of new rabies-related lyssaviruses over the past decades has resulted in an explosion of lyssavirus species globally, with little knowledge regarding their potential risk to human and animal lives [191].

Continued research into the pathogenicity of rabies and its interplay with the immune system can facilitate new therapeutic options for the treatment of other ailments, including those infections that are able to cross the blood–brain barrier. The use of rabies viral particles has been paramount to an improved understanding of the nervous system and synaptic processes [192,193] and can remain a useful tool for further study. RABV is also being used as a target-specific vector for the brain and spinal cord as a delivery means for vaccines and other therapeutic options. The means by which the RABV proteins interact with the immune system and tight junction proteins will provide valuable insights into delivering therapeutics and gene related therapies into immune privileged areas.

Lastly, the availability of rabies therapeutics that are cheaper to produce and also pose fewer ethical considerations—when compared with RIG—will facilitate the achievement of the global target for canine-mediated human rabies elimination. The requirement for post-exposure prophylaxis and rabies therapeutics, although reduced, will not cease when canine-mediated human rabies is eliminated as continued human exposures are likely to occur due to the presence of other reservoir species. However, with the elimination of canine rabies, the demand for PEP will decrease, thus resulting in a decreased production and availability of PEP and a likely increase in costs as it will no longer be produced on a mass scale. With a cheaper and easier alternative to the current regime, PEP can be produced locally and can remain available for any future exposures to rabid animals.

In this review, we have highlighted important immune factors that influence the infection, spread and pathogenicity of rabies. These factors will be important considerations to research groups looking to develop new and cheaper therapeutics for rabies, and for those research groups that intend to use RABV as a mechanism to study the nervous system and immune privileged areas of the host. Canine rabies elimination should remain a priority to reach the global target of elimination by 2030 and therefore the mass vaccination of dogs should be prioritized. Concurrently, an improved understanding of the disease, its pathogenesis, and its complex interactions with the immune system, as well as the potential for cheaper and improved therapeutics, should be used to facilitate human rabies elimination, the treatment of emerging rabies and rabies-related lyssavirus variants, as well as further knowledge of the nervous system and other associated diseases.
